# Smenamides A and B, Chlorinated Peptide/Polyketide Hybrids Containing a Dolapyrrolidinone Unit from the Caribbean Sponge *Smenospongia aurea*. Evaluation of Their Role as Leads in Antitumor Drug Research

**DOI:** 10.3390/md11114451

**Published:** 2013-11-08

**Authors:** Roberta Teta, Elena Irollo, Gerardo Della Sala, Giuseppe Pirozzi, Alfonso Mangoni, Valeria Costantino

**Affiliations:** 1 The NeaNat Group, Dipartimento di Farmacia, Università degli Studi di Napoli Federico II, via D. Montesano 49, Napoli 80131, Italy; E-Mails: roberta.teta@unina.it (R.T.); gerardo.dellasala@unina.it (G.S.); alfonso.mangoni@unina.it (A.M.); 2 Department of Experimental Oncology, Istituto Nazionale Tumori Fondazione “G. Pascale”, Via M. Semmola, Napoli 80131, Italy; E-Mails: e.irollo@istitutotumori.na.it (E.I.); g.pirozzi@istitutotumori.na.it (G.P.)

**Keywords:** *Smenospongia aurea*, marine natural products, peptide/polyketides hybrids, structure elucidation, antitumor activity

## Abstract

An in-depth study of the secondary metabolites contained in the Caribbean sponge *Smenospongia aurea* led to the isolation of smenamide A (**1**) and B (**2**), hybrid peptide/polyketide compounds containing a dolapyrrolidinone unit. Their structures were elucidated using high-resolution ESI-MS/MS and homo- and heteronuclear 2D NMR experiments. Structures of smenamides suggested that they are products of the cyanobacterial metabolism, and 16S rRNA metagenomic analysis detected *Synechococcus spongiarum* as the only cyanobacterium present in *S. aurea*. Smenamides showed potent cytotoxic activity at nanomolar levels on lung cancer Calu-1 cells, which for compound **1** is exerted through a clear pro-apoptotic mechanism. This makes smenamides promising leads for antitumor drug design.

## 1. Introduction

As part of a broad screening program going on at the NeaNat research group focused on the study of sponge natural product chemistry in order to discover novel lead molecules for anticancer [1] and anti-inflammatory [2] drug design, we performed an in-depth study of the chemistry of the lipophilic extract of the sponge *Smenospongia aurea* (order Dictyoceratida, family Thorectidae), collected by Scuba along the coast of Little Inagua (Bahamas Islands).

Marine sponges of the family Thorectida, and particularly those of the genus *Smenospongia*, are a well-known sources of many interesting secondary metabolites. Among them, it is worth mentioning the large number of indole alkaloids [3], often mono- or polybrominated, but terpenes [4], and sesquiterpene quinones and hydroquinones [5,6,7] have also been found. Despite the extensive work by different research teams on the chemistry of this genus, there are very few reports on *S.* *aurea*, partly because many papers lack identification at the specie level.

**Figure 1:**
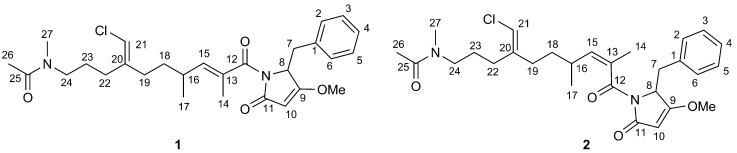
Structures of smenamide A (**1**) and B (**2**).

A preliminary NMR and MS examination of the lipophilic extract of *S.* *aurea* revealed the presence of many known compounds, including some brominated alkaloids related to aplysynopsin [8,9]. LC-MS-based examination of the extract revealed the presence of two isomeric compounds whose mass did not fit any known natural product. The compounds turned out to be hybrid peptide/polyketide natural products, which were called smenamide A (**1**) and B (**2**) (Figure 1). Here, we report the isolation and structure elucidation of smenamides, along with an examination of their remarkable cytotoxic activity.

## 2. Results and Discussion

### 2.1. Structural Elucidation

*Smenospongia aurea* from the coast of Little Inagua (Bahamas Islands) was extracted with MeOH/CHCl_3_ mixtures. The organic extract was separated by flash chromatography on RP-18 silica gel, followed by repeated reversed- and normal-phase chromatography, to give pure compounds **1** (15 µg) and **2** (8 µg). In spite of the very small amounts of the isolated compounds, it was possible to obtain a full set of homonuclear and heteronuclear two-dimensional NMR spectra (COSY, TOSCY, ROESY, HSQC, and HMBC) for both compounds **1** and **2**, which allowed the complete assignment of their planar structure. All the ^13^C chemical shift could be assigned using the 2D spectra, and therefore 1D ^13^C NMR spectra were not recorded.

The positive ion mode high-resolution ESI mass spectrum of smenamide A (**1**) (Supplementary Figure S1) displayed [M + H]^+^ and [M + Na]^+^ pseudomolecular ion peak at *m/z* 501.2508 and 523.2326, respectively. Both ions showed intense (32%) M + 2 isotope peaks, suggesting the presence of one atom of chlorine, and were indicative of the molecular formula C_28_H_37_ClN_2_O_4_ (calcd. 501.2515 for C_28_H_38_ClN_2_O_4_ and 523.2334 for C_28_H_37_ClN_2_O_4_Na). The peak at *m/z* 487.2557 ([M − HCl + Na]^+^) in the HRMS/MS spectrum confirmed the presence of chlorine in the molecule.

In the ^1^H NMR spectrum of compound **1** (Supplementary Figure S3), most resonances were split into two signals in approximately 1:1 ratio. This suggested a conformational equilibrium, slow in the NMR time scale, but fast enough to prevent separation of the two conformers. A confirmation of the presence of conformational equilibrium was sought in the ROESY spectrum (Supplementary Figure S4). In this experiment, in fact, besides cross peaks originating from NOE, cross peaks originating from chemical exchange are also present, which can be easily distinguished from the former in that they have opposite phase. As expected, clear exchange cross peaks were observed between the methyl singlets at δ 3.03 and 2.88 (H_3_-27) and the methine singlets at δ 5.93 and 5.97 (H-21) (all the chemical shifts in the following discussion will refer to one conformer; see Table 1 for the complete NMR assignment).

**Table 1:** NMR data of smenamide A (**1**) (700 MHz, CD_3_OD).

		*Z*-Conformer		*E*-Conformer			
Position		δ_H_ [Mult., *J* (Hz)]	δ_C_ [Mult.]	δ_H_ [Mult., *J* (Hz)]	δ_C_ [Mult.]	COSY	HMBC
1		−	135.6 (C)	−	135.6 (C)		
2/6		6.99 (m)	130.8 (CH)	6.99 (m)	130.8 (CH)	3/5	4
3/5		7.23 (ovl)	129.4 (CH)	7.23 (ovl)	129.4 (CH)	2/6	1
4		7.23 (ovl)	128.3 (CH)	7.23 (ovl)	128.3 (CH)	2/6	
7	a	3.37 (ovl)	34.8 (CH_2_)	3.37 (ovl)	34.8 (CH_2_)	7b, 8	1, 2/6, 8, 9
	b	3.19 (m)		3.19 (m)		7a, 8	2/6
8		5.02 (ovl)	60.5 (CH)	5.02 (ovl)	60.5 (CH)	7a, 7b	
9		−	179.5 (C)	−	179.5 (C)		
10		5.04 (br. s)	95.5 (CH)	5.02 (br. s)	95.5 (CH)		8, 11
11		−	170.7 (C)	−	170.7 (C)		
12		−	172.3 (C)	−	172.2 (C)		
13		−	132.1 (C)	−	132.1 (C)		
14		1.77 (d, 1.5)	13.7 (CH_3_)	1.78 (d, 1.5)	13.7 (CH_3_)	15	12, 13, 15
15		5.36 (br. d, 10.2)	144.1 (CH)	5.36 (br. d, 10.2)	144.1 (CH)	14, 16	
16		2.45 (m)	33.4 (CH)	2.48 (m)	33.4 (CH)	15, 17, 18a	
17		0.98 (d, 6.5)	20.4 (CH_3_)	1.00 (d, 6.5)	20.6 (CH_3_)	16	15, 18, 19
18	a	1.51 (ovl)	36.1 (CH_2_)	1.52 (ovl)	35.9 (CH_2_)	16, 19a, 19b	19
	b	1.28 (ovl)		1.30 (ovl)		19a, 19b	
19	a	2.19 (ovl)	33.2 (CH_2_)	2.23 (ovl)	33.2 (CH_2_)	18a, 18b, 19b, 21	20, 21
	b	2.06 (ovl)		2.05 (ovl)		18a, 18b, 19a, 21	20
20		−	143.1 (C)	−	142.8 (C)		
21		5.93 (br. s)	113.9 (CH)	5.97 (br. s)	114.1 (CH)	19a, 19b	20, 22
22	a	2.22 (m)	28.1 (CH_2_)	2.26 (m)	28.0 (CH_2_)	22b, 23	20, 21
	b	2.15 (m)		2.18 (m)		22a, 23	20
23		1.64 (m)	25.9 (CH_2_)	1.70 (m)	26.6 (CH_2_)	22a, 22b, 24	
24		3.36 (ovl)	48.6 (CH_2_)	3.33 (ovl)	51.5 (CH_2_)	23	22, 23, 25, 27
25		−	172.9 (C)	−	172.7 (C)		
26		2.08 (s)	21.7 (CH_3_)	2.07 (s)	21.1 (CH_3_)	27	25
27		3.03 (s)	36.6 (CH_3_)	2.88 (s)	33.7 (CH_3_)	26	24, 25
OMe		3.97 (s)	59.7 (CH_3_)	3.97 (s)	59.7 (CH_3_)		9

The molecular formula and the general features of the ^1^H NMR spectrum, including five aromatic protons of a monosubstituted benzene ring, three olefinic protons, and five methyl groups (one *O*-methyl, one *N*-methyl, one acetyl methyl, one olefinic methyl, and one aliphatic methyl) suggested smenamide A (**1**) to be the product of a PKS/NRPS pathway.

The *N*-methyl group (CH_3_-27, δ_H_ 3.03, δ_C_ 36.6) and the acetyl methyl group (CH_3_-26, δ_H_ 2.08, δ_C_ 21.7) showed correlation peaks in the HMBC spectrum with the same carbonyl carbon atom at δ 172.9, suggesting an *N*-methylacetamido function. This was confirmed by the long-range coupling between the two methyls through the amide bond [10] evidenced by the COSY spectrum. The amide carbonyl was also coupled with the methylene protons at δ 3.36 (H_2_-24), which were part of the trimethylene chain C-22/C-24 whose protons were assigned through the COSY spectrum.

Protons on the part structure between C-12 and C-19 are part of the same spin system, and each of them (including the olefinic methyl protons H_3_-14) showed a correlation peak with the methyl protons at δ 0.98 (H_3_-17) in the TOCSY spectrum. They were then sequentially assigned using the COSY spectrum, while the HMBC couplings of H_3_-14 with C12, C-13, and C15, and that of H_3_-17 with C-15, C-16, and C-18 definitely confirmed the structure of this part of the molecule. The configuration of the double bond at position 13 was defined as *E* on the basis of the ROESY correlation peaks between H_3_-14 and H-16, and of the absence of any correlation peak between H_3_-14 and H-15.

The two partial structures defined so far were connected through a trisubstituted double bond, as demonstrated by the HMBC correlation peaks (Supplementary Figure S5) of protons at C-19 and C-22 with the methine carbon atom at δ 113.9 and the non-protonated carbon atom at δ 143.1, as well as by the allylic coupling of protons at C-19 with the olefinic proton H-21 (δ 5.93). The low-field chemical shift of H-21, together with the high-field chemical shift of C-21 (δ 113.9) [10], suggested the chlorine atom required by the molecular formula to be linked to C-21. The *Z* configuration of the double bond was proven by the ROESY correlation peak between H-21 and H-19a (δ 2.06).

**Figure 2:**
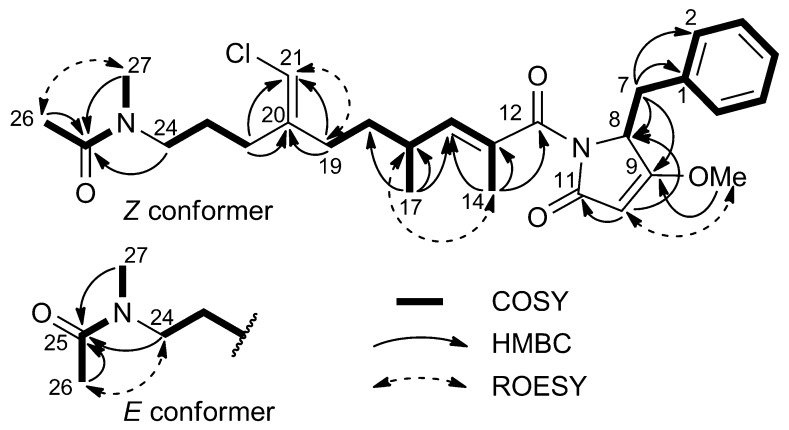
Most significant correlations provided by the COSY, HMBC, and ROESY 2D NMR spectra of smenamide A (**1**).

The remaining part of the molecule was composed of a benzyl group, clearly recognizable from the NMR data, which was linked to a heterocycle identified as 4-methoxy-1*H*-pyrrol-2(5*H*)-one on the basis of the ROESY correlation peak of the metoxy group (δ 3.97) with H-10 (δ 5.04), and the proton-carbon long-range coupling of H-10 with C-8 and C-11, and those of the protons at C-7 (δ 3.37 and 3.19) with C-8 and C-9 (Figure 2). This benzyl pyrrolone unit is known as dolapyrrolidinone (Dpy) and is the terminal unit of the cytotoxic depsipeptide dolastatin-15 [11]. The ^13^C chemical shifts of Dpy carbon atoms in smenamide A matched well the corresponding ones in dolastatin-15, with a maximum difference of 1.5 ppm, further supporting this structural assignment.

**Figure 3:**
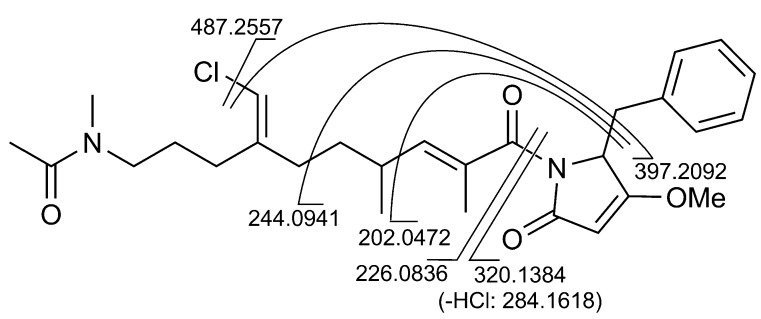
Fragment ions observed in the high-resolution ESI MS/MS spectrum of smenamide A (**1**).

Finally, a high resolution MS/MS spectrum (Supplementary Figure S2) was recorded using the sodiated pseudomolecular ion peak of **1** at *m*/*z* 523 as the parent ion. All the observed fragment ions (Figure 3) were in full agreement with the proposed structure.

After the structure of smenamide A (**1**) was determined, and its ^1^H and ^13^C NMR spectra completely assigned, the origin of the observed conformational equilibrium could be identified as a *E*/*Z* inversion occurring at the amide bond of the terminal *N*-methylacetamido group, because the largest chemical shift differences between the two conformers were observed in this part of the molecule. In addition, the *Z* conformer showed a strong correlation peak between H_3_-26 and H_3_-27 in the ROESY spectrum, absent for the *E* conformer, which showed instead a correlation peak between H_3_-26 and H_2_-24 (Figure 2).

Smenamide A (**1**) contains two stereogenic centers at C-8 and C-16. The configuration of them could not be determined, because this would have required degradation methods, while we preferred to use the very limited amounts of compound available for biological assays.

Smenamide B (**2**) was isomeric with smenamide A, as shown by the high-resolution ESI mass spectrum (Supplementary Figure S6; [M + H]^+^ ion at *m/z* 501.2505 and [M + Na]^+^ ion at and 523.2320). The general features of the ^1^H NMR spectrum (Supplementary Figure S8) were the same (including the conformational equilibrium between two conformers), with slightly different chemical shifts for all signals. Also the correlation peaks in the COSY, TOCSY, HSQC, and HMBC 2D NMR spectra were very similar (Supplementary Table S1), and the fragment ions in the MS/MS spectrum (Supplementary Figure S7) were identical. This led to the conclusion that smenamide B is a stereoisomer of smenamide A. The difference between the two compounds was located in the configuration of the double bond at C-13, which was determined as *Z* in smenamide B on the basis of the correlation peak between H_3_-14 (δ 1.79) and H-15 (δ 5.13) observed in the ROESY spectrum.

There are no close analogs of smenamides among hybrid peptide/polyketide natural products, but smenamides share structural features with several known compounds. The dolapyrrolidinone terminus is the same as in dolastatin-15, a depsipeptide isolated from the sea hare *Dolabella auricolaria* [11] but believed to be of likely cyanobacterial origin [12], and in other cyanobacterial metabolites, namely mycapolyols A–F [13] and belamide A [14]. The presence of a pendent vinyl chloride in the middle of the polyketide chain is a feature shared with jamaicamides from the cyanobacterium *Lyngbia majuscula* [15]. In contrast, the *N*-methylacetamido starting unit has never been found in a polyketide/peptide hybrid, and is only found in a few natural products, all biogenetically unrelated with smenamides (remarkably, one of them has been found in a sponge of the genus *Smenospongia* [16]). Therefore, all the compounds related to smenamide A and B either were isolated from cyanobacteria or are of cyanobacterial origin.

### 2.2. 16S rRNA Metagenomic Analysis

The structural motifs of smenamide A and B strongly suggest that they are a product of the cyanobacterial metabolism. In addition, smenamides have been found in comparable amounts in three different specimens of *S. aurea* collected in different years and geographical areas, suggesting their symbiotic, rather than dietary, origin.

Therefore, the composition of the cyanobacterial community in *S. aurea* was examined by biomolecular analysis. A PCR experiment performed using the metagenome of *S. aurea* as template and the cyanobacterial-specific 16S rRNA primers CYA106F and CYA781R(a) or CYA781R(b) [17] produced clear bands at the expected length of about 670 bp. A 16S rRNA gene library was then constructed from the amplicons and 24 clones were sequenced. Except for three sequences not related to cyanobacteria, all the cyanobacterial sequences were identical at a 99% sequence identity threshold, and assigned to *Candidatus Synechococcus spongiarum*. This is a lineage of uncultured unicellular sponge-specific symbiotic cyanobacteria, which are the most prevalent and widespread symbiotic cyanobacteria in tropical and temperate reef sponges [18].

The detection of *S. spongiarum* as the only cyanobacterium present in *S. aurea* raises the question of whether it may be responsible for the biosynthesis of smenamides. Maybe because of its widespread occurrence, *S. spongiarum* has never been considered among the possible producers of natural products, which are generally sought among more specialists symbionts. However, horizontal gene transfer of PKS and NPRS biosynthetic gene clusters across phylogenetically divergent bacteria is not uncommon [19]. In addition, several distinct clades of *S. spongiarum* are present in marine sponges, and many of them are clearly host-specific [18,20].

It can be speculated that different clades of *S. spongiarum* may contain different horizontally transferred biosynthetic gene clusters, and therefore may be able to produce the metabolites characteristic of each species. Given the unavailability of the genome of any *S. spongiarum*, this is only a speculation, but it can be the rationale for future work on these cyanobacteria.

### 2.3. *In Vitro* Evaluation of Cytotoxic Activity of Smenamides

Peptide/polyketide hybrid natural products of cyanobacterial origin often show cytotoxic activity to cancer cells, which range from moderate to extraordinary high, as in the case of dolastatin-10 [21]. It is remarkable that dolastatin-10 is one of the still few marine natural products that has advanced to clinics: in 2011, over twenty years after its discovery, its semisynthetic analogue auristatin E, in combination with a monoclonal antibody, has been approved as a treatment for Hodgkins lymphoma [22].

These considerations led us to test the possible antitumor activity of smenamides. Because non-small-cell lung cancer (NSCLC) represents nearly 85% of primary malignant tumors, the biological activity of smenamides was tested on an NSCLC cell line, namely Calu-1.

**Figure 4:**
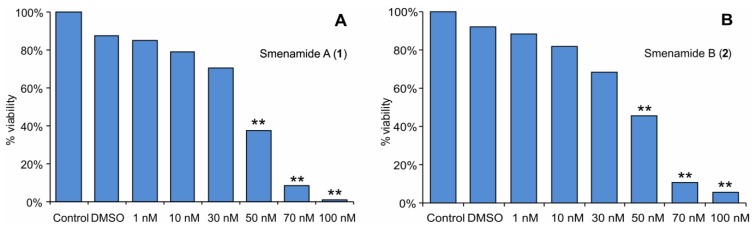
Evaluation by MTT assay of Calu-1 cell viability after 72 h of treatment with (**A**) compound **1** and (**B**) compound **2**. ** *P* < 0.0005.

First, the cytotoxic activity of compounds **1** and **2** on Calu-1 was analyzed by MTT cell viability assays. After 72 h of treatment, no significant cytotoxic effect was observed at concentrations up 30 nM. In contrast, a remarkable cytotoxic effect was observed at concentrations 50 nM and higher (Figure 4). The estimated IC50 values of the two compounds were nearly identical, 48 nM for **1** and 49 nM for **2.**

**Figure 5:**
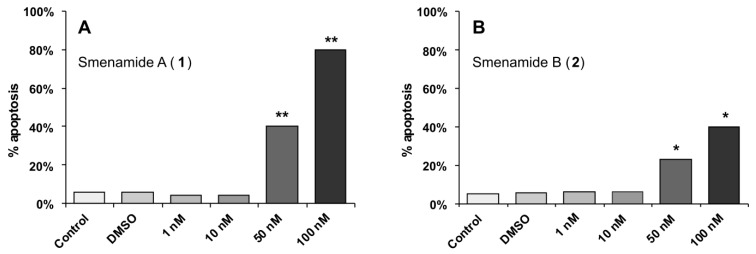
Evaluation of pro-apoptotic activity of smenamides using the Annexin-V FITC/PI assay. (**A**) The percentage of apoptosis for cells treated for 72 h with compound **1** at 1, 10, 50, and 100 nM was, respectively, 4%, 4%, 40%, and 80%; the remaining cells remained viable. (**B**) The percentage of apoptosis for cells treated for 72 h with compound **2** at 1, 10, 50, and 100 nM was, respectively, 6%, 6%, 23%, 40%; the remaining cells remained viable except at concentration of 100 nM, where 47% of cells were necrotic. ** *P* < 0.0005, * *P* < 0.001.

Next, Annexin-V FITC/PI assays were performed to evaluate whether the cytotoxic activity of compounds **1** and **2** was related to apoptosis induction. Annexin binds to phosphatidylserine when it is exposed on the outer leaflet of the plasma membrane, as it happens during early apoptosis, while propidium iodide (PI) binds to DNA only when the cells is dead. Therefore, this method allows apoptotic cells to be distinguished both from viable cells and from necrotic cells. The two compounds produced remarkably different results in this assay (Figure 5). Compound **1** appears to exert its cytotoxic activity through a clear pro-apoptotic mechanism, with a dose-dependent increase of apoptotic cells and little or no necrotic cells. For compound **2**, the percentage of apoptotic cells was much lower, and at 100 nM necrotic cells were present in high percentage (47%). This different behavior, which is not revealed by the standard MTT cytotoxicity assay, seems to suggest different mechanisms of action for the two molecules. This could be related to their different overall shape dictated by the configuration of the C-13/C-15 double bond, located close to the middle of the molecule.

A preliminary examination of the biological activity of smenamides on other types of solid tumors (such as breast, ovary and melanoma) led to similar results (data not shown).

## 3. Experimental Section

### 3.1. General Experimental Procedures

High-resolution ESI-MS spectra were performed on a Thermo LTQ Orbitrap XL mass spectrometer. The spectra were recorded by infusion into the ESI source using MeOH as the solvent. CD spectra were recorded on a Jasco J-710 spectrophotometer using a 1-cm cell. NMR spectra were determined on Varian Unity Inova spectrometers at 700 MHz; chemical shifts were referenced to the residual solvent signal (CD_3_OD: δ_H_ 3.31, δ_C_ 49.00). For an accurate measurement of the coupling constants, the one-dimensional ^1^H NMR spectra were transformed at 64 K points (digital resolution: 0.09 Hz). Through-space ^1^H connectivities were evidenced using a ROESY experiment with a mixing time of 450 ms. The HSQC spectra (acquisition times: 13 h for **1** and 22 h for **2**) were optimized for ^1^*J*_CH_ = 142 Hz, and the HMBC experiments (acquisition times: 61 h for **1** and 50 h for **2**) for ^2,3^*J*_CH_ = 8.3 Hz. High performance liquid chromatography (HPLC) separations were achieved on a Varian Prostar 210 apparatus equipped with a Varian Prostar 325 UV-Vis detector.

### 3.2. Collection, Extraction, and Isolation

A specimen of *Smenospongia aurea* was collected by Scuba along the coast of Little Inagua (Bahamas Islands) during the 2013 Pawlik expedition and immediately identified onboard following the information reported in http://www.spongeguide.com. The sample was frozen immediately after collection and stored at −20 °C until extraction. For biomolecular analysis, a small portion of the specimen was stored in five volumes of RNAlater (Life Technologies, Carlsbad, CA, USA) stabilization solution and kept at −20 °C until used. The sponge (1046 g wet weight) was homogenized and extracted with MeOH (5 × 5 L), MeOH and CHCl_3_ in different ratios (2:1, 1:1, 1:2) and then with CHCl_3_ (6 × 5 L). The MeOH extracts were partitioned between H_2_O and *n*-BuOH; the BuOH layer was combined with the CHCl_3_ extracts and concentrated *in vacuo*.

The total organic extract (12.25 g) was chromatographed on a column packed with RP-18 silica gel. A fraction eluted with MeOH/H_2_O (9:1, 340 mg) was subjected to reversed-phase HPLC separation on an RP-18 column [MeOH/H_2_O (8:2), Luna C18, 250 × 10 mm, 10 μm; λ = 280 nm], thus affording a fraction (*t_R_* = 10 min) containing compound **1** and a fraction (*t_R_* = 8 min) containing the compound **2**. The former fraction was subjected to normal-phase HPLC on an SiO_2_ column [*n*-Hex/*iso*-PrOH (8:2), Luna Silica, 250 × 4.6 mm, 5 μm; λ = 250 nm], which gave 15 µg of pure compound **1**.

The fraction containing compound **2** was first subjected to HPLC in the same condition as for **1**; final purification was achieved by HPLC on an analytical RP-18 column using CH_3_CN/H_2_O as eluent [ACN/H_2_O (7:3), Luna C18—250 × 4.6 mm, 5 μm; λ = 215 nm], to obtain pure compound **2** (8 µg).

LC-MS analysis of the crude extract of two additional specimen of *S. aurea*, collected in 2007 at Sweeting Cay (Grand Bahama Island) and in 2008 at Little San Salvador (Bahamas Islands), respectively, showed them to contain similar amounts of compounds **1** and **2**.

### 3.3. Smenamide A *(**1**)*

Colorless amorphous solid, HRESIMS (positive ion mode, MeOH) *m*/*z* 523.2326 ([M + Na]^+^, C_28_H_37_ClN_2_O_4_Na^+^, calcd. 532.2334), *m*/*z* 501.2509 ([M + Na]^+^, C_28_H_38_ClN_2_O_4_^+^, calcd. 501.2515); MS isotope pattern: M (100%), M + 1 (32%, calcd. 31.5%), M + 2 (37%, calcd. 36.0%), M + 3 (10%, calcd. 10.6%,); HRESIMS/MS (parent ion *m*/*z* 523.23, C_28_H_37_ClN_2_O_4_Na^+^): *m*/*z* 487.2557 (C_28_H_36_N_2_O_4_Na^+^, calcd. 487.2567), 397.2092 (C_21_H_30_N_2_O_4_Na^+^, calcd. 397.2098), 320.1384 (C_16_H_24_ClNO_2_Na^+^, calcd. 320.1388), 284.1618 (C_16_H_23_NO_2_Na^+^, calcd. 284.1621), 244.0941 (C_12_H_15_NO_3_Na^+^, calcd. 244.0944), 226.0836 (C_12_H_13_NO_2_Na^+^, calcd. 226.0838), 202.0472 (C_9_H_9_NO_3_Na^+^, calcd. 226.0475); ^1^H and ^13^C NMR: Table 1; UV (MeOH): λ_max_(ε) 287 nm (8200), 246 nm (46000), 225 nm (92000); CD (MeOH): λ_max_(Δε) 238 (+33), 219 (−30).

### 3.4. Smenamide B *(**2**)*

Colorless amorphous solid, HRESIMS (positive ion mode, MeOH) *m*/*z* 523.2320 ([M + Na]^+^, calcd. for C_28_H_37_ClN_2_O_4_Na^+^ 532.2334), *m*/*z* 501.2505 ([M + Na]^+^, calcd. for C_28_H_38_ClN_2_O_4_^+^ 501.2515); MS isotope pattern: M (100%), M + 1 (31%, calcd. 31.5%), M + 2 (36%, calcd. 36.0%), M + 3 (11%, calcd. 10.6%,); HRESIMS/MS (parent ion *m*/*z* 523.23, C_28_H_37_ClN_2_O_4_Na^+^): *m*/*z* 487.25567 (C_28_H_36_N_2_O_4_Na^+^, calcd. 487.2567), 397.2091 (C_21_H_30_N_2_O_4_Na^+^, calcd. 397.2098), 320.1383 (C_16_H_24_ClNO_2_Na^+^, calcd. 320.1388), 284.1617 (C_16_H_23_NO_2_Na^+^, calcd. 284.1621), 244.0941 (C_12_H_15_NO_3_Na^+^, calcd. 244.0944), 226.0835 (C_12_H_13_NO_2_Na^+^, calcd. 226.0838), 202.0471 (C_9_H_9_NO_3_Na^+^, calcd. 226.0475); ^1^H and ^13^C NMR: Table 1; ^1^H and ^13^C NMR: Supplementary Table S1; UV (MeOH): λ_max_(ε) 287 nm (15000), 248 nm (42000), 225 nm (84000); CD (MeOH): λ_max_(Δε) 237 (+7.3).

### 3.5. DNA Isolation

To ~40 mg of frozen sponge (in RNA later) 700 µL of lysis buffer I (200 mM Tris-Cl, 50 mM EDTA, 1.4 M NaCl, 2% CTAB, 0.5% PVP, all in milliQ^®^-H_2_O) and 50 µL lysozime (20 mg/mL) were added and incubated at 37 °C for 1 h in a thermomixer (1400 rpm). After addition of 2.8 µL β-mercaptoethanol, 70 µL 10% SDS, 2 µL RNase A (100 mg/mL), and 40 µL proteinase K (10 mg/mL) the tube was incubated at 55 °C for a further hour in a thermomixer (1400 rpm). At this time, the microcentrifuge tube was spun 4 min at 5000 rpm. The clear middle phase was transferred to a new microcentrifuge tube containing 750 µL CHCl_3_ and centrifuged 10 min at 15,000 rpm. After two further CHCl_3_ washes, the supernatant was transferred to a new microcentrifuge tube containing 750 µL of 70% aqueous isopropyl alcohol containing 10% (v/v) 3 M NaOAc (pH 5.5) at room temperature. The precipitated DNA was spun down at top speed for 20 min (4 °C), washed with ice-cold ethanol, dried and dissolved in ~100 µL of water.

### 3.6. PCR Amplification and Clone Library Construction

Metagenomic DNA from S. aurea was used for PCR amplification with the 16S rRNA cyanobacterial specific primers CYA106F (5′-CGGACGGGTGAGTAACGCGTGA-3′), CYA781R(a) (5′-GACTACTGGGGTATCTAATCCCATT-3′) and CYA781R(b) (5′-GACTACAGGGGTATCTAATCCCTTT-3′). Polymerase chain reaction was carried out under the following conditions: initial denaturation at 94 °C for 45 s, followed by 33 cycles of 94 °C for 1 min, 60 °C/64 °C for 1 min and 72 °C for 45 s, with a final extension 72 °C for 10 min. The reaction mixture (25 µL) contained: 14.65 µL H_2_O, 0.5 µL DMSO, 1.5 µL dNTP (10 mM), 2.5 µL Taq buffer advanced (Eppendorf, Hamburg, Germany), 2.5 µL forward primer CYA106F (10 µM), 2.5 µL reverse primer CYA781R(a) or CYA781R(b) (10 µM), 0.35 µL RBC Taq DNA polymerase (5 U/µL, RBC Bioscience, New Taipei City, Taiwan), 0.5 µL DNA. The PCR products of the expected size (~670 bp) were purified from the agarose gel using QIAquick gel ex kit (Qiagen, Venlo, The Netherlands), subcloned via T/A cloning into pBluescriptII SK(+) (Stratagene California, La Jolla, CA, USA), and transferred to *E. coli* XL1 Blue MRF' (Stratagene California, La Jolla, CA, USA) electrocompetent cells. After blue white screening, plasmid preps were sent to GATC Biotech AG (Köln, Germany) for single read sequencing using the T7 primer.

### 3.7. Cell Culture

Calu-1 cell line was purchased from the American Type Culture Collection (ATCC). Cell line was grown in Iscove’s modified Dulbecco’s medium (IMDM) (Gibco, Life Technologies, Carlsbad, CA, USA), supplemented with 10% FBS (Gibco, Life Technologies, Carlsbad, CA, USA), 100 units/mL Penicillin plus 100 µg/mL Streptomycin (Lonza, Basel, Switzerland), 250 mg/mL Amphotericine B (Lonza, Basel, Switzerland). Cell cultures were incubated at 37 °C in a humidified 5% CO_2_ atmosphere. Cells were harvested at 80% confluence to perform further examination.

### 3.8. MTT Assay

Cell viability was determined by 3-(4,5-dimethylthiazol-2-yl)-2,5-diphenyltetrazolium bromide (MTT) assay according to the manufacturer’s instructions. Briefly, cells were plated in 96-well plates, at a density of 3 × 10^3^ cells/well, in a total volume of 100 μL of complete medium. After treatment for 72 h with compounds **1** and **2** at different concentrations (1, 10, 30, 50, 70, and 100 nM), MTT (5 mg/mL in PBS) was added to each well, and the cells were incubated for 4 h at 37 °C. Dimethylsulfoxide (DMSO) was added to each well after removal of the supernatant, and the plates were shaken to dissolve formazan. The absorbance reading of each well was determined using a computer-controlled microtiter plate reader (Bio-Rad, Hercules, CA, USA) at a wavelength of 540 nm. All experiments were performed in triplicate for each condition.

The optical density of the cells incubated with culture medium alone was taken as 100% viability. Differences between groups were determined by analysis of variance (ANOVA) and were considered statistically significant at *p* < 0.05. The IC50 values were determined by nonlinear regression analysis using a 4-parameter logistic equation.

### 3.9. Apoptosis assay

Apoptosis was assessed using the Annexin-V FITC kit (Milteny Biotec, Bergisch Gladbach, Germany) according to manufacturer’s instructions. Briefly, cells were plated in 6-well plates at a density of 100 × 10^5^ cells/well and were treated for 72 h with compounds **1** and **2** at concentrations 1, 10, 50, and 100 nM. After treatment cells were recovered, washed with Binding Buffer (BB) and centrifuged. Annexin-V FITC (10 μL) was added to the pellets, which were then incubated in the dark for 15 min at room temperature. Next, cells were washed and re-suspended in BB, and propidium iodide solution (5 μL) was added immediately prior to analysis by flow citometry. The percentage of apoptosis was analyzed by Diva Software using a Becton Dickinson FACS ARIA II.

### 3.10. Accession Codes

The cyanobacterial 16S rRNA partial sequences from metagenomic analysis were deposited into GenBank under the accession numbers KF692286 through KF692298 (primers CYA106F/CYA781R(a)), and KF692299 through KF692309 (primers CYA106F/CYA781R(b)).

## 4. Conclusion

The discovery of smenamides further expands the catalog of highly bioactive marine natural products, and can potentially provide new templates for the development of antitumor drugs.

Smenamide A (**1**) and B (**2**) are two isomeric hybrid peptide/polyketide compounds containing a dolapyrrolidinone unit, which are present in low concentrations in the marine sponge *S. aurea*. In spite of the very little amounts of the isolated compounds (15 μg for **1** and 8 μg for **2**), their planar structures were completely determined by spectroscopic means.

The structural features of smenamides suggest them to be products of cyanobacterial metabolism; the only cyanobaterial 16S rRNA sequences detected in the metagenome of *S. aurea* belong to *Synechococcus spongiarum*, a symbiotic cyanobacterium widespread in tropical and temperate reef sponges.

Smenamides showed potent cytotoxic activity at nanomolar level on lung cancer Calu-1 cells, which is exerted through a clear pro-apoptotic mechanism only for compound **1**. Therefore, while displaying similar IC50 inhibitory values for human Calu-1 lung cancer cells, compounds **1** and **2** do not seem to exert their respective activity through the same mechanism. Further studies are in progress to gain a better insight into the antitumor activity of smenamides. It is important to point out that the widespread diffusion of *S. aurea* will make it possible to isolate higher amounts of smenamides, making them amenable for further development as antitumor compounds.

## Supplementary Files

Supplementary File 1 Supplementary Information (PDF, 405 KB)
