# Supplementary material for: Smenamides A and B, Chlorinated Peptide/Polyketide Hybrids Containing a Dolapyrrolidinone Unit from the Caribbean Sponge Smenospongia aurea. Evaluation of Their Role as Leads in Antitumor Drug Research
Source: Mar Drugs. 2013 Nov 8;11(11):4451–63. doi: 10.3390/md11114451 (PMC3853738; doi:10.3390/md11114451)

Supplementary Information

Table S1. NMR data of smenamide A (**1**) (CD_3OD).
Figure S1. Positive ion mode high-resolution ESI mass spectrum of smenamide A (**1**).
Figure S2. Positive ion mode high-resolution ESI MS/MS spectrum of smenamide A (**1**).
Figure S3. ^{1}H-NMR spectrum of smenamide A (**1**) (700 MHz, CD_3OD).
Figure S4. ROESY spectrum of smenamide A (**1**) (700 MHz, CD_3OD).
Figure S5. HMBC spectrum of smenamide A (**1**) (700 MHz, CD_3OD).
Figure S6. Positive ion mode high-resolution ESI mass spectrum of smenamide B (**2**).
Figure S7. Positive ion mode high-resolution ESI MS/MS spectrum of smenamide B (**2**).
Figure S8. ^{1}H-NMR spectrum of smenamide B (**2**) (700 MHz, CD_3OD).

Table S1. NMR data of smenamide B (**2**) (700 MHz, CD_3OD).

Position		***Z*-Conformer**		***E*-Conformer**			
		**δ_H [Mult., *J* (Hz)]**	**δ_C [Mult.]**	**δ_H [Mult., *J* (Hz)]**	**δ_C [Mult.]**	**COSY**	**HMBC**
1		−	135.5 (C)	−	135.5 (C)		
2/6		7.00 (m)	131.0 (CH)	7.00 (m)	131.0 (CH)	3/5, 7b	4, 6/2, 7
3/5		7.22 (ovl)	129.4 (CH)	7.22 (ovl)	129.4 (CH)	2/6	1
4		7.21 (ovl)	128.3 (CH)	7.21 (ovl)	128.3 (CH)	2/6	
7	a	3.54 (m)	35.3 (CH_2)	3.54 (m)	35.3 (CH_2)	7b, 8	1, 2/6, 8, 9
	b	3.20 (ovl)		3.20 (ovl)		2/6, 7a, 8	1, 2/6, 8
8		4.95 (m)	60.7 (CH)	4.95 (m)	60.7 (CH)	7a, 7b	
9		−	180.7 (C)	−	180.7 (C)		
10		5.05 (br. s)	95.6 (CH)	5.02 (br. s)	95.6 (CH)		8, 11
11		−	171.0 (C)	−	171.0 (C)		
12		−	171.5 (C)	−	171.5 (C)		
13		−	134.2 (C)	−	134.2 (C)		
14		1.788 (d, 1.5)	20.2 (CH_3)	1.784 (d, 1.5)	20.2 (CH_3)	15	12, 13, 15
15		5.13 (br. d, 10.1)	137.2 (CH)	5.13 (br. d, 10.1)	137.2 (CH)	14, 16	12, 14
16		2.10	35.4 (CH)	2.13	35.4 (CH)	15, 17, 18a, 18b	
17		0.95 (d, 7.1)	21.2 (CH_3)	0.96 (d, 7.1)	21.2 (CH_3)	16	15, 18, 19
18	a	1.37 (ovl)	36.1 (CH_2)	1.39 (ovl)	35.9 (CH_2)	16, 18b, 19a, 19b	
	b	1.31 (ovl)		1.32 (ovl)		16, 18a, 19a	
19	a	2.08 (ovl)	33.3 (CH_2)	2.10 (ovl)	33.3 (CH_2)	18a, 18b, 19b, 21	20, 21
	b	1.92 (ovl)		1.93 (ovl)		18a, 19a	
20		−	143.2 (C)	−	143.2 (C)		
21		5.81 (br. s)	113.9 (CH)	5.86 (br. s)	113.9 (CH)	19a	
22		2.13 (ovl)	28.2 (CH_2)	2.18 (ovl)	28.0 (CH_2)	23	20, 21
23		1.61 (m)	25.9 (CH_2)	1.70 (m)	26.6 (CH_2)	22, 24	22, 24
24		3.34 (ovl)	48.4 (CH_2)	3.34 (ovl)	51.4 (CH_2)	23	22, 23, 25, 27
25		−	173.4 (C)	−	173.2 (C)		
26		2.07 (s)	21.3 (CH_3)	2.08 (s)	21.1 (CH_3)	27	25
27		3.02 (s)	36.5 (CH_3)	2.90 (s)	33.6 (CH_3)	26	24, 25
OMe		3.95 (s)	59.7 (CH_3)	3.94 (s)	59.7 (CH_3)		9

Figure S1. Positive ion mode high-resolution ESI MS spectrum of smenamide A (**1**).

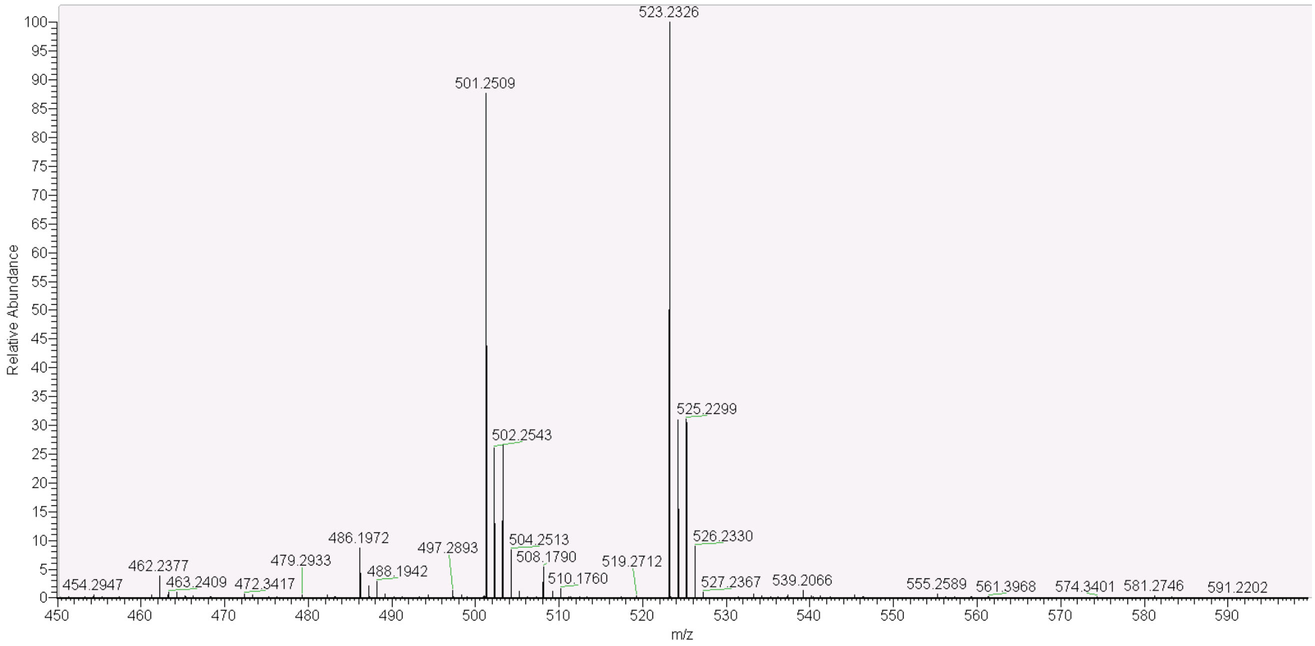

Figure S2. Positive ion mode high-resolution ESI MS/MS spectrum of smenamide A (**1**), parent ion at *m*/*z* 523.23.

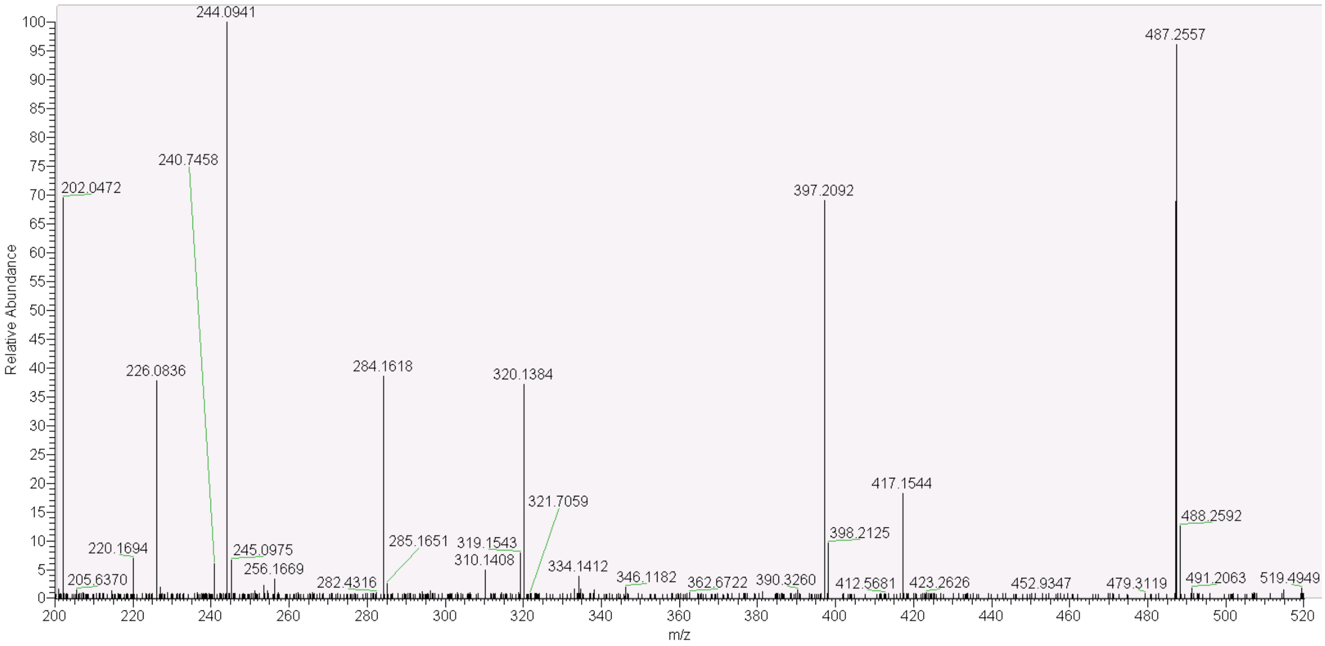

Figure S3. ^{1}H NMR spectrum of smenamide A (**1**) (CD_3OD, 700 MHz).

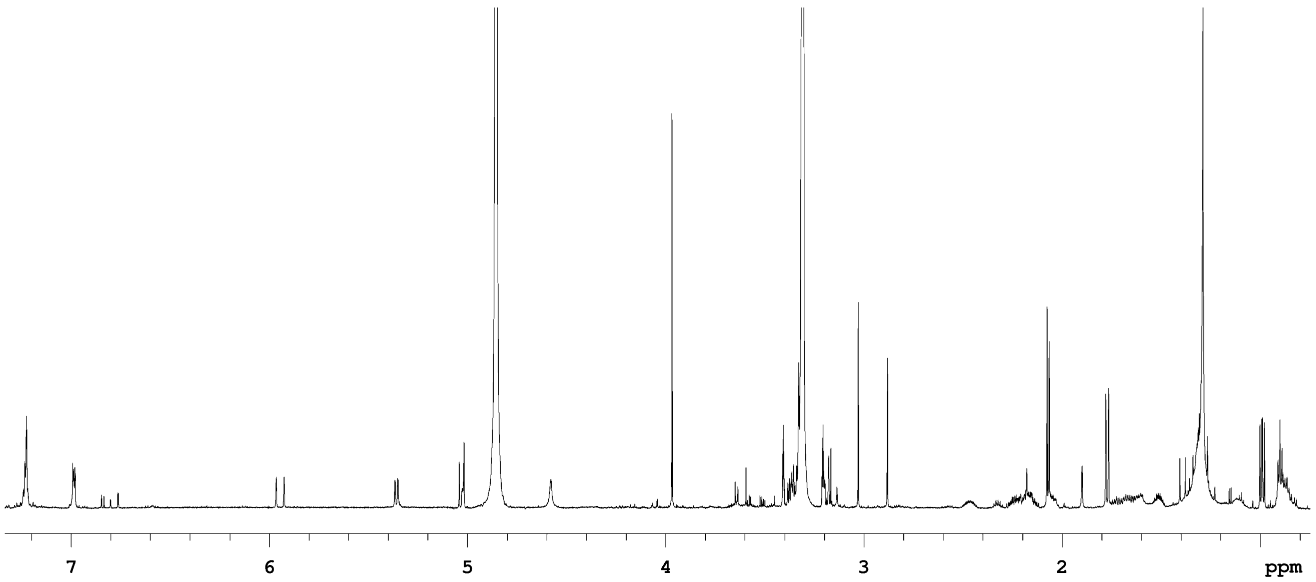

Figure S4. ROESY spectrum of smenamide A (**1**) (CD_3OD, 700 MHz).

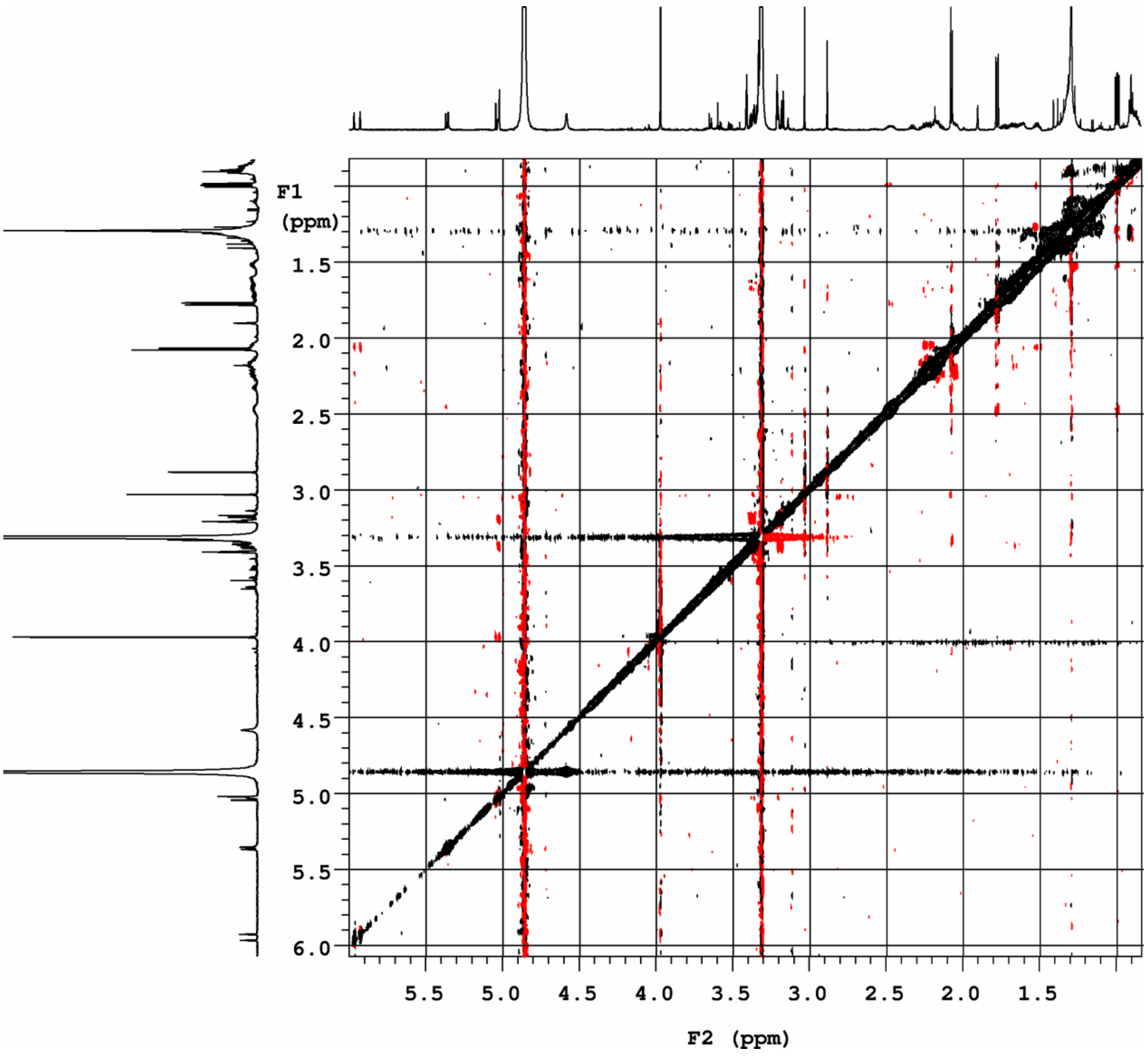

Figure S5. HMBC spectrum of smenamide A (**1**) (CD_3OD, 700 MHz).

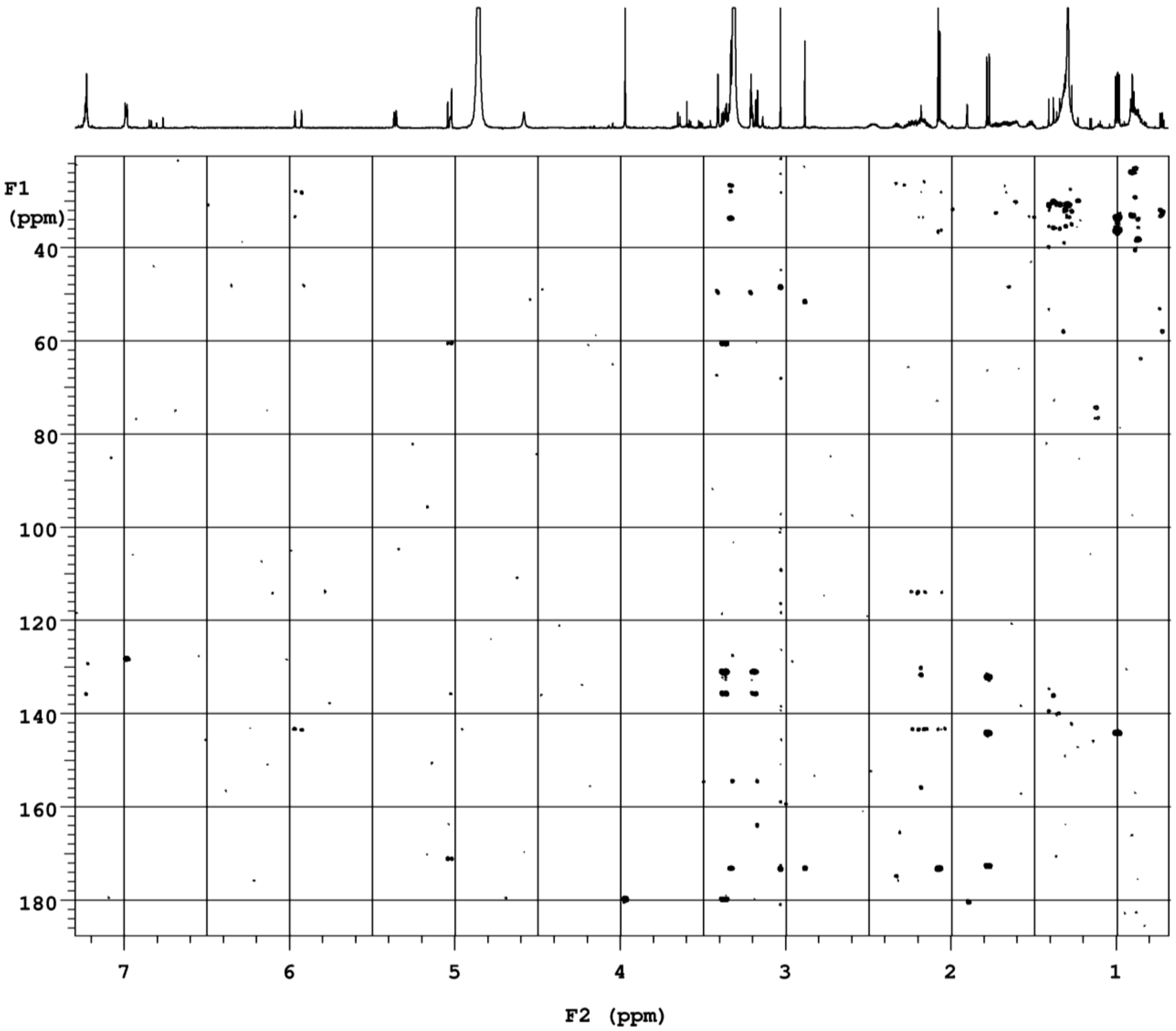

Figure S6. Positive ion mode high-resolution ESI MS spectrum of smenamide B (**2**).

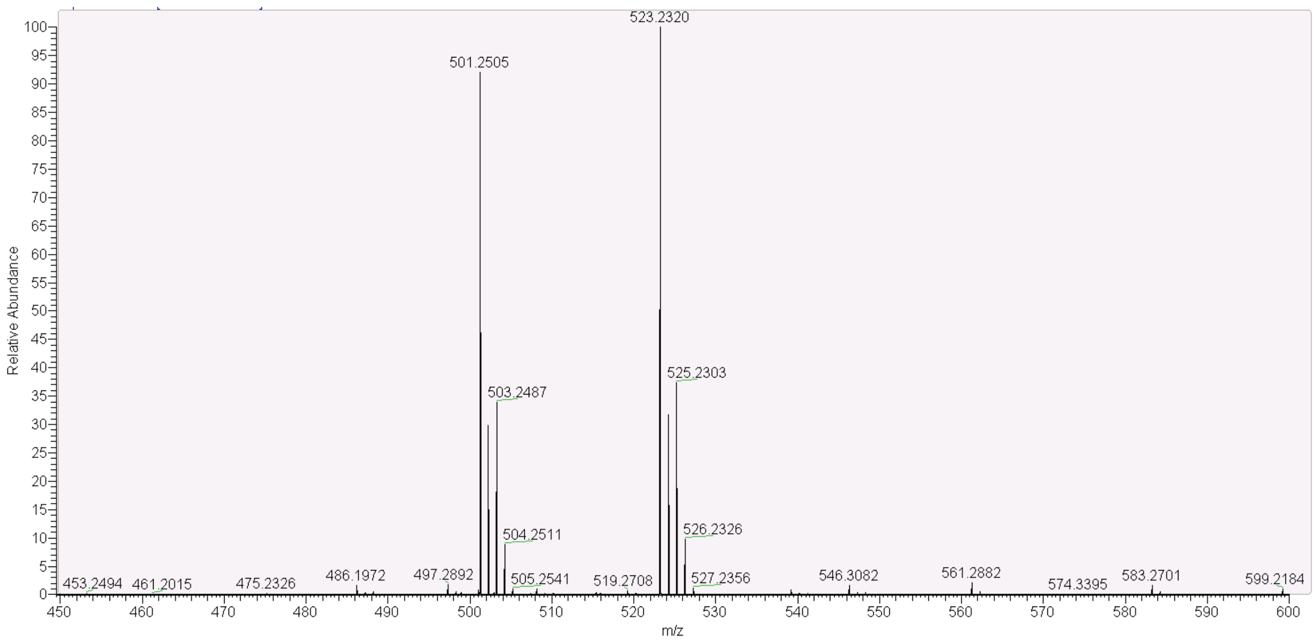

Figure S7. Positive-ion high-resolution ESI MS/MS spectrum of smenamide B (**2**), parent ion at *m*/*z* 523.23.

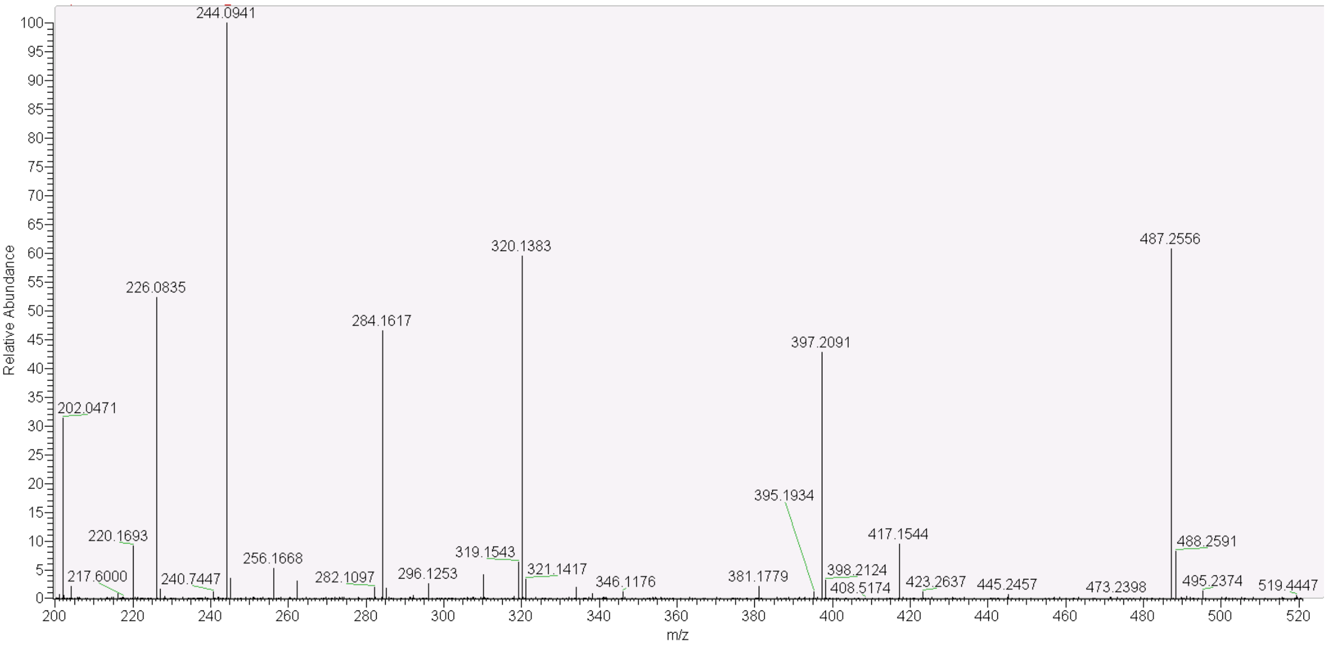

Figure S8. ^{1}H NMR spectrum of smenamide B (**2**) (CD_3OD, 700 MHz).

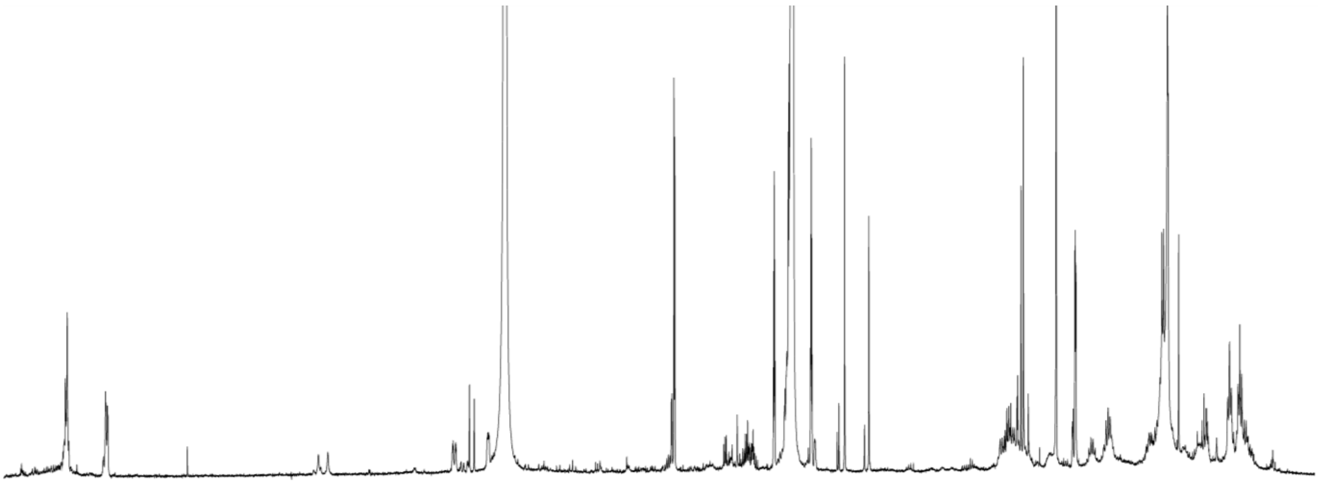

Supplement: Supplementary File 1 — Supplementary Information (PDF, 405 KB) [file marinedrugs-11-04451-s001.pdf]
